# Dairy intake and cardiovascular diseases risk factors: a cross-sectional study on Iranian obese and overweight women

**DOI:** 10.1186/s12889-024-19232-z

**Published:** 2024-07-15

**Authors:** Dorsa Hosseininasab, Farideh Shiraseb, Rasool Ghaffarian-Ensaf, Shabnam Hosseini, Alessandra da Silva, Mohammad Mahdi Hajinasab, Vaughn W. Barry, Barbora de Courten, Khadijeh Mirzaei

**Affiliations:** 1 Department of Nutrition, Science and Research Branch, Islamic Azad University, Tehran, Iran; 2 Department of Community Nutrition, School of Nutritional Sciences and Dietetics, Tehran University of Medical Sciences (TUMS), P.O. Box: 14155-6117, Tehran, Iran; 3 School of Human Nutrition, McGill University, Montreal, QC Canada; 4 Universidade Federal de Viçosa, Viçosa, Brazil; 5 Department of Nutrition, Electronic Health and Statistics Surveillance Research Center, Science and Research Branch, Islamic Azad University, Tehran, Iran; 6 Health and Human Performance, Middle Tennessee State University, Murfreesboro, TN USA; 7 Department of Medicine, School of Clinical Sciences, Monash University, Melbourne, VIC 3168 Australia; 8 School of Health and Biomedical Sciences, RMIT University, Melbourne, VIC 3085 Australia

**Keywords:** Dairy, Cardiovascular diseases, Obesity, Overweight, Women

## Abstract

**Objective:**

Atherosclerotic cardiovascular disease (ASCVD) is one of the leading causes of death worldwide. Dietary interventions can directly affect several ASCVD risk factors. This study aimed to assess an association between dairy consumption and the odds of ASCVD and its risk factors in women with overweight and obesity.

**Methods:**

The present cross-sectional study was conducted on 390 Iranian women aged 18–48 years and body mass index (BMI) ≥ 25 kg/m². Dairy consumption was assessed using a 147-item food frequency questionnaire. Participants were divided into tertiles based on their dairy consumption with 130 (33.3%) women in each category.

**Results:**

The participants had an average age of 36.73 ± 9.18 years, and the mean BMI was 31.28 ± 4.30 kg/m^2^. In the unadjusted model, individuals in the third tertile of dairy consumption had 0.79 times lower odds of ASCVD compared to those in the first tertile (OR: 0.21; 95% Confidence Interval (CI): 0.11, 0.41; P-value = 0.001). Additionally, we observed a significant inverse relationship between higher dairy intake and adiposity markers, blood pressure, and Triglyceride glucose-body mass index (TyG-BMI).

**Conclusion:**

The study revealed a negative association between dairy intake and the risk of ASCVD but this association diminished after adjusting for confounding factors. It also found a negative association between dairy consumption with BMI, fat mass index, body fat, blood pressure, and TyG-BMI.

**Supplementary Information:**

The online version contains supplementary material available at 10.1186/s12889-024-19232-z.

## Introduction

As per the World Health Organization (WHO), atherosclerotic cardiovascular disease (ASCVD) stands as the primary global cause of death, accounting for approximately 17.9 million fatalities in 2016, with 85% attributed to myocardial infarction (MI) and stroke [1]. In Tehran, cardiovascular disease (CVD) is responsible for over 40% of mortality [2]. Recognized risk factors for ASCVD encompass unhealthy lifestyle behaviors such as tobacco use, unhealthy dietary patterns, physical inactivity, obesity, and consumption of alcohol [3]. Lifestyle interventions, specifically dietary interventions and increased physical activity, directly mitigate risk factors for ASCVD, including dyslipidemia, insulin resistance, hypertension, and abdominal obesity [4–6].

Obesity stands as a significant contributor to the onset and advancement of ASCVD [7]. This prevalent health issue is on the rise in both developed and developing nations, due to unhealthy dietary habits and lack of physical activity [8–14]. Additionally, it is recognized that, beyond overall adiposity, measures such as waist circumference (WC) and waist-to-height ratio (WHtR) are even more strongly associated with ASCVD and its risk factors [15–18].

Smoking can cause disturbances in blood vessel dilation, increase oxidative stress, and increase inflammation [19, 20]. It has also been shown that smoking can increase LDL and cholesterol [21, 22]. Emily Banks et al. showed that smoking increases the risk of CVD and doubles the risk of myocardial infarction and heart failure [23]. The average global per capita consumption of alcohol has increased from 5.5 L in 2005 to 6.4 L in 2016 and is predicted to reach 7.6 L by 2025 [24]. Alcohol consumption not only leads to social issues but also increases mortality and chronic diseases in different societies [25]. Joaquim Fernández-Solà and Jürgen Rehm has shown that alcohol consumption is associated with a significant increase in the risk of Cardiomyopathy [26, 27]. Studies have also shown that alcohol consumption can increase the risk of atrial fibrillation [28–30].

Dietary interventions play a crucial role in preventing and treating various chronic diseases, including CVD [31]. While numerous studies highlight the impact of nutrients on preventing conditions like hypertension, hypercholesterolemia, and stroke [32–35], there has been limited focus on the specific contributions of food, particularly dairy products [36–38]. Dairy products, a widely consumed and diverse food group globally [39], contribute significantly to calcium and protein intake [40]. Developed nations consume milk at an average per capita level of around 172 g while developing nations only consume around 71 g [41].

Some dairy products have been associated with positive effects on certain CVD risk factors in some studies. For instance, a meta-analysis of cohort studies on dairy intake and blood pressure fluctuations over time showed a reduced risk of hypertension with increasing milk consumption, while the effect on overall CVD risk was neutral [42]. Ding et al. and Sellem et al. found no significant association between dairy consumption and CVD risk factors [43, 44].

On the other hand, the Dietary Approaches to Stop Hypertension (DASH) trial found a significant association between blood pressure and dairy intake [44]. Another systematic review and meta-analysis of randomized controlled trials (RCT) showed that cheese consumption increased LDL cholesterol concentration [45].

Given the worldwide prevalence of CVD and its contradictory results with regards to some dairy products consumption, the present study aims to assess the association between dairy consumption and lifetime odds of ASCVD and its risk factors among women with overweight and obesity in Iran.

## Methods

### Study participants

This cross-sectional study was performed in health centers in Tehran, Iran. This formula n = (([Z1-α – Z1-β) ×√1 − r2]/r)2 + 2 α = 0.05 β = 0.95 *r* = 0.20) was used to calculate the sample size of 360. Considering the possibility of leaving 5% of the participants the final sample size was calculated to be 390.

Since the evaluated population was very large and scattered, the multi-stage random sampling process was used. A total of 20 health centers were randomly selected from all health centers affiliated with the Tehran University of Medical Sciences. Then, the participants who met the criteria were randomly recruited for this study. Inclusion criteria encompassed women aged 18 to 48 years, classified as overweight or obese (overweight: BMI of 25–29.9 kg/m^2^, obesity: BMI of ≥ 30 kg/m^2^), who willingly agreed to participate. Participants were required to have no acute or chronic infections, no history of type 2 diabetes mellitus, CVD, polycystic ovary syndrome (PCOS), stroke, non-alcoholic fatty liver disease (NAFLD), inflammatory diseases, hypertension, cancer, and thyroid disease. Exclusion criteria included the use of medications influencing weight, blood pressure, blood lipoproteins, and blood glucose, as well as the use of alcohol and/or any kind of supplements. Pregnant, lactating, and menopause women, individuals with outrageous energy consumption lower than 800 kcal/day and/or more than 4200 kcal/day [46], and those who left more than 70 food items of the food frequency questionnaire (FFQ) were also excluded. The study protocol received approval from the ethics committee of Tehran University of Medical Sciences (TUMS) under the reference number of IR.TUMS.VCR.REC.1398.142, and all participants provided written informed consent before participation.

### Dietary assessment

The participants’ regular dietary intakes were assessed using a validated and reliable semi-quantitative FFQ that included 147 food items [47]. A trained nutritionist filled out the FFQs through face-to-face interviews with participants. Individuals were asked to report their consumption for each food item per day, week, month, or year. Subsequently, portion sizes were converted to grams using Iranian household measures [48]. Nutrient and energy intakes were evaluated using NUTRITIONIST-IV (version 7.0; N Squared Computing, Salem, OR, USA) [48].

Seven categories of dairy variables were established based on daily intake of dairy products: total dairy, low-fat dairy, high-fat dairy, total milk, total yogurt, cheese, and fermented dairy. Total dairy encompassed all dairy products except for ice cream and butter. Low-fat dairy included low-fat milk and yogurt (with < 2% total fat content), as well as low-fat cheese (< 20% total fat content). High-fat dairy comprised high-fat milk, high-fat yogurt (≥ 2% total fat content), high-fat cheese (≥ 20% total fat content), and chocolate milk. The definition of total dairy excluded ice cream and butter due to their high sugar and fat content, respectively. Total milk included both low-fat and high-fat milk, as well as chocolate milk. Total yogurt comprised all yogurt types, while cheese included both low-fat and high-fat variations (traditional cheese, feta cheese, cream cheese, and other cheeses). Fermented dairy covered all yogurt and cheese types [49].

### Biochemical markers

All participants underwent blood sample collection in the morning following a 10–12 h fast. The serum was centrifuged and then stored at -80^ºC^. Standard protocols were employed to measure serum levels of fasting blood glucose (FBG), total cholesterol (TC), low and high-density lipoprotein cholesterol (LDL, HDL), triglycerides (TG), alanine transaminase (ALT), aspartate transaminase (AST), high-sensitivity C-reactive protein (hs-CRP), monocyte chemoattractant protein-1 (MCP-1), interleukin 1 beta (IL-1β), galectin-3 (Gal-3), and transforming growth factor-beta (TGF-β). Plasminogen activator inhibitor-1 (PAI-1) was assessed in triplicate using Human PAI-1*96 T ELISA kit from Crystal Company. All measurements were conducted at the Nutrition and Biochemistry laboratory of the School of Nutritional Sciences and Dietetics utilizing assessment kits from Pars Azmoon (Pars Azmoon Inc. Tehran, Iran). Insulin resistance homeostatic model assessment (HOMA-IR) was calculated using the formula: [fasting plasma glucose (mmol/l) × fasting plasma insulin (mIU/l)]/22.5 [50].

### Lifetime ASCVD risk, atherogenic index of plasma (AIP), and lipid ratio assessment

The lifetime risk of ASCVD was calculated using an ASCVD Risk Estimator Algorithm, published in 2013, ACC/AHA guidelines, and updated in 2017 [51, 52]. Participants below the age of 20 years were excluded from this calculation because the algorithm specifically provides lifetime risk estimates for individuals aged 20 to 59 years. Various indices were computed to assess atherogenic factors: AIP, Castelli index-1, Castelli index-2, and atherogenic coefficient (AC) were determined using the formulas log (TG/HDL), TC/HDL, LDL/HDL, and (TC-HDL) /LDL, respectively [53]. CHOLINDEX was calculated as LDL-HDL when TG levels were < 400 mg/dl and LDL-HDL + 1/5 of TG when TG levels were ≥400 mg/dL [53]. The triglyceride-glucose index (TyG index) was computed as Ln (FBG (mg/dl) ×TG (mg/dl)/2) [54]. TyG-WC and TyG-BMI are defined as TyG×WC and TyG×BMI, respectively [55].

### Anthropometric measurement

Anthropometric measurements for all participants were conducted at the Nutrition and Biochemistry Laboratory of the School of Nutrition and Dietetics, TUMS. Weight was determined using a digital scale (Seca, Hamburg, Germany) while participants wore thin clothing without shoes, with an accuracy of approximately 0.1 kg. Height was measured using a Seca stadiometer with an accuracy of about 0.1 cm. WC was assessed at the smallest distance between the lower end of the sternum (xiphoid process) and the umbilicus, and hip circumference was measured at the largest hip area. WHtR was also calculated. All measurements were carried out by a trained nutritionist.

### Body composition assessment

Body composition indicators, including fat mass, fat mass index, fat-free mass, fat-free mass index, visceral adipose tissue, BMI, WHtR, and trunk fat were assessed using the In-body 770 scanner, a multi-frequency bioelectrical impedance analyzer (In-body Co., Seoul, Korea). This tetrapolar bioimpedance analyzer utilizes electrodes from hand to foot. To minimize potential measurement variations, participants were instructed to avoid vigorous exercise and excessive intake of fluids or food before undergoing the body composition assessments. These evaluations were conducted in the morning following urination, while participants were in a fasting state.

### Assessment of other covariates

Blood pressure was assessed on the left arm after a resting period of at least 10 min by a skilled physician using a standard sphygmomanometer (Omron, Germany, Europe). Demographic information was collected through questionnaires covering age, education level (illiterate, diploma, bachelor and higher), marital status (single, married), income level (poor, moderate, good), employment status (employed, unemployed), specific diet, medical history, medication, and supplementation. These questionnaires were administered by trained nutritionists. Physical activity was evaluated using the short version of validated international physical activity questionnaire (IPAQ). This assessment was computed as metabolic equivalent hours per week (METs hours/week) [56]. Trained interviewers asked participants to report on all the vigorous- and moderate-intensity activities over the last seven days. The duration and frequency of activity days were multiplied to compute the overall activity, and the sum of the scores represented the total physical activity per week.

### Statistical analysis

The normality of quantitative variables was assessed using the Kolmogorov – Smirnov test, and the normality of all variables was confirmed (*P* > 0.05). One-way analysis of variance (ANOVA) was used to determine the mean and standard deviation (SD) of quantitative variables according to dairy tertiles. Additionally, analysis of covariance (ANCOVA) was employed to evaluate the mean of quantitative variables according to dairy tertiles, adjusting for potential confounders. The frequency of categorical variables according to dairy tertiles was examined using Pearson’s chi-square test, and the results were expressed as n (%). The association between dairy tertiles and CVD risk factors was assessed through the generalized linear model (GLM) and the outputs of this test were presented as beta (β) and 95% CI values. Furthermore, the association between dairy tertiles and the odds of ASCVD was examined using binary logistic regression, with results presented as odds ratios (OR) and 95% CI. In model 1, the variables included were age, energy intake, BMI, physical activity, and no use of weight loss supplements. Model 2 included the variables from Model 1 plus vegetables, meats, and refined grains. All analyses were conducted using SPSS 26, and P-values < 0.05 were considered statistically significant.

## Results

This study enrolled 390 women characterized by overweight and obesity. The participants had a mean age of 36.73 ± 9.18 years and a BMI of 31.28 ± 4.30 kg/m^2^. 70% of the participants were married, and 98.5% were employed. In terms of economic status, 46.7% of participants fell into the category of moderate economic status, and 47.2% had completed a bachelor’s degree or higher. In the crude model and after controlling confounders including age, BMI, energy intake and physical activity, there was not any significant mean difference among quantitative variables (*p* > 0.05). Significant difference was detected across dairy tertiles for supplement intake before (*p* = 0.002) and after adjustment (*p* = 0.046) (Table 1).

**Table 1:** General characteristics among tertiles of dairy consumption in obese and overweight women (*n* = 390)

Variables		Dairy Tertile			*P*-value	*P*-value*
		T1	T2	T3		
		< 257.042	257.042-420.041	> 420.041		
Quantitative variables						
Age (year)		37.90 ± 9.85	36.33 ± 9.17	35.8 ± 8.43	0.201	0.220
Body profile						
Weight (kg)		79.55 ± 12.01	81.76 ± 12.87	82.21 ± 12.56	0.174	0.614
Height (cm)		160.06 ± 6.00	161.36 ± 5.77	161.97 ± 5.76	**0.028**	0.545
PA (MET- min-week)		1348.48 ± 2946.39	1014.93 ± 1298.96	1230.91 ± 1700.35	0.600	0.524
Categorical variable						
Education status	Illiterate	1 [25]	2 [50]	1 [25]	0.327	0.756
	Under diploma	15 (32.6)	10 (21.7)	21 (45.7)		
	Diploma	52 (35.1)	55 (37.2)	41 (27.7)		
	Bachelor and higher	58 (31.5)	62 (33.7)	64 (34.8)		
Job status	Employed	128 (33.3)	127 (33.1)	129 (33.6)	0.607	0.527
	unemployed	0 (0)	1 [50]	1 [50]		
Marital status	Married	32 (29.4)	41 (37.6)	36 [33]	0.542	0.545
	Non-married	94 (34.4)	88 (32.2)	91 (33.3)		
Economic status	Poor	29 [33]	31 (35.2)	28 (31.8)	0.337	0.106
	Moderate	66 (36.3)	62 (34.1)	54 (29.7)		
	Good	31 (29.2)	31 (29.2)	44 (41.5)		
Supplement intake	Yes	45 (28.5)	46 (29.1)	67 (42.4)	**0.002**	**0.046**
	No	67 (38.3)	65 (37.1)	43 (24.6)		

PA: Physical activity

Values are represented as means ± SD and categorical n (%)

P-value with unadjusted (crude)

P-values were obtained through ANCOVA and one-way ANOVA analysis

P value *: Adjusted for age, BMI, physical activity, and energy intake. BMI considered as collinear for weight variable

Bold valued indicates presence of statistical significance (P-value < 0.05) or marginally significance (p-value = 0.06 and 0.07)

### Energy, nutrients, and food groups across tertiles of dairy consumption

Table 2 presents the dietary intakes of women based on the tertiles of dairy consumption. After adjusting for energy intake, the consumption of protein, cholesterol, saturated fatty acid, monounsaturated fatty acid, polyunsaturated fatty acid, linoleic fatty acid, vitamins A, D, E, B2, B5, B6, B9, B12, sodium, potassium, magnesium, calcium, phosphorus, zinc, and fiber were significantly different amongst the tertiles of dairy intake. In addition, among food groups, only refined grains, vegetables, and meat consumption demonstrated a significant increase according to dairy tertiles, after controlling for the energy intake.

**Table 2:** Dietary intake of women with overweight and obesity according to tertiles of dairy consumption (*n* = 390)

Energy, nutrients, and food groups	Dairy tertiles			*P*-value	*p*-value*
	T1 (*n* = 130)	T2 (*n* = 130)	T3 (*n* = 130)		
	< 257.042 g	257.042–420.041 g	> 420.041 g		
Energy intake (kcal/d)	2292.51 ± 695.72	2566.16 ± 769.45	3046.65 ± 779.08	**< 0.001**	-
Protein (g/d)	73.18 ± 22.34	88.39 ± 25.732	112.47 ± 32.21	**< 0.001**	**< 0.001**
Carbohydrate (g/d)	324.60 ± 109.20	366.11 ± 120.137	427.72 ± 122.42	**< 0.001**	0.400
Fat (g/d)	85.30 ± 35.30	91.33 ± 34.062	108.88 ± 32.08	**< 0.001**	0.140
Cholesterol (g/d)	217.63 ± 98.25	252.43 ± 100.011	322.15 ± 115.43	**< 0.001**	**< 0.001**
SFA (mg/d)	22.98 ± 10.10	26.85 ± 9.092	35.38 ± 11.70	**< 0.001**	**< 0.001**
MUFA (mg/d)	29.38 ± 13.65	30.88 ± 13.301	35.80 ± 10.86	**< 0.001**	**0.037**
PUFA (mg/d)	19.91 ± 10.91	19.39 ± 9.752	20.97 ± 7.80	0.397	**< 0.001**
Linoleic (g/d)	17.56 ± 10.31	16.86 ± 9.181	17.81 ± 7.28	0.680	**< 0.001**
Linolenic (g/d)	1.09 ± 0.73	1.14 ± 0.576	1.39 ± 0.64	**0.001**	0.638
EPA (g/d)	0.02 ± 0.03	0.02 ± 0.036	0.03 ± 0.04	0.305	0.618
DHA (g/d)	0.08 ± 0.10	0.09 ± 0.111	0.11 ± 0.12	0.220	0.583
TFA (g/d)	0.00 ± 0.00	0.00 ± 0.002	0.00 ± 0.00	0.170	0.289
Vitamin A (RAE-mcg/d)	600.21 ± 341.34	684.91 ± 290.42	1004.77 ± 452.75	**< 0.001**	**< 0.001**
Vitamin D(mcg/d)	0.96 ± 0.574	1.64 ± 0.87	3.27 ± 1.84	**< 0.001**	**0.001**
Vitamin E (mg/L)	17.11 ± 10.354	16.53 ± 9.41	17.45 ± 7.13	0.706	**0.001**
Vitamin k (mcg/d)	240.30 ± 263.855	274.12 ± 260.22	348.29 ± 337.89	**0.009**	0.457
Vitamin B1 (mg/d)	1.88 ± 0.706	2.13 ± 0.69	2.41 ± 0.71	**< 0.001**	0.106
Vitamin B2 (mg/d)	1.65 ± 0.513	2.19 ± 0.69	2.98 ± 0.80	**< 0.001**	**< 0.001**
Vitamin B3 (mg/d)	23.02 ± 8.057	25.67 ± 8.74	30.41 ± 11.76	**< 0.001**	0.996
Vitamin B5 (mg/d)	4.97 ± 1.515	6.12 ± 1.59	8.27 ± 2.58	**< 0.001**	**< 0.001**
Vitamin B6 (mg/d)	1.81 ± 0.602	2.11 ± 0.67	2.66 ± 0.73	**< 0.001**	**< 0.001**
Vitamin B9 (mg/d)	555.09 ± 185.152	622.12 ± 190.48	685.06 ± 182.14	**< 0.001**	**0.047**
Vitamin B12 (mg/d)	2.80 ± 1.515	3.98 ± 1.37	6.23 ± 2.88	**< 0.001**	**< 0.001**
Sodium (mg/d)	4142.93 ± 1532.630	4463.45 ± 1916.28	4851.45 ± 1747.49	**0.005**	**0.043**
Potassium (mg/d)	3611.75 ± 1400.777	4297.71 ± 1533.62	5631.64 ± 1600.02	**< 0.001**	**< 0.001**
Magnesium (mg/d)	400.74 ± 144.426	462.33 ± 165.22	565.23 ± 163.42	**< 0.001**	**0.009**
Calcium (mg/d)	880.42 ± 340.516	1214.47 ± 369.94	1714.85 ± 503.94	**< 0.001**	**< 0.001**
Phosphorus	1273.48 ± 374.283	1620.92 ± 430.67	2133.15 ± 505.96	**< 0.001**	**< 0.001**
Iron (mg/d)	23.74 ± 19.747	26.36 ± 19.45	29.29 ± 23.15	0.101	0.379
Zinc (mg/d)	10.80 ± 3.663	13.06 ± 4.27	16.39 ± 4.92	**< 0.001**	**< 0.001**
Selenium (µg/d)	111.80 ± 45.830	125.50 ± 46.01	142.32 ± 52.43	**< 0.001**	0.321
Chromium (mg/d)	0.11 ± 0.096	0.11 ± 0.10	0.12 ± 0.10	0.449	**0.075**
Fiber (g/d)	43.65 ± 21.546	46.63 ± 19.98	51.94 ± 21.78	**0.006**	**0.040**
Caffeine (g/d)	146.29 ± 195.186	158.21 ± 123.49	155.87 ± 115.80	0.793	0.629
**Food groups**					
Whole grains (g/d)	7.20 ± 10.99	7.31 ± 9.57	8.25 ± 10.66	0.740	0.982
Fruits (g/d)	445.39 ± 294.03	498.09 ± 333.62	636.23 ± 355.47	**< 0.001**	0.455
Vegetables (g/d)	364.65 ± 256.38	395.77 ± 226.42	531.83 ± 274.18	**< 0.001**	**0.005**
Nuts (g/d)	14.06 ± 19.00	11.67 ± 11.70	17.10 ± 16.60	**0.063**	0.160
Legumes (g/d)	45.15 ± 33.96	56.29 ± 45.22	56.82 ± 43.10	0.086	0.315
Refined grains (g/d)	409.93 ± 215.68	448.83 ± 194.37	438.87 ± 245.76	0.454	**0.003**
Tea and coffee	753.28 ± 1118.17	728.56 ± 560.60	743.87 ± 446.30	0.975	0.326
Eggs (g/d)	21.16 ± 12.89	20.37 ± 13.22	23.40 ± 16.02	0.299	0.683
Processed food (g/d)	22.16 ± 24.13	22.61 ± 25.03	27.68 ± 29.99	0.269	0.887
Meat (g/d)	51.57 ± 32.39	58.20 ± 36.62	82.17 ± 66.98	**< 0.001**	**0.039**

Values are represented as means ± SD. P = values were obtained through One-way ANOVA. P-values* were obtained through ANCOVA adjusted by energy intake. Bold valued indicates presence of statistical significance (P-value < 0.05) or marginally significance (p-value = 0.06 and 0.07)

Abbreviation: DHA: Docosahexaenoic acid; EPA: Eicosapentaenoic acid; MUFA: Monounsaturated fatty acid; PUFA: Polyunsaturated fatty acid; SFA: Saturated Fatty Acid; TFA: Trans fatty acid

### CVD risk factors across tertiles of dairy consumption

Table 3 illustrates the anthropometric measurements, body composition indicators, and clinical markers across dairy consumption tertiles. After controlling for confounders in model 2, we observed that participants with higher dairy intake had a significant lower mean values of BMI, fat mass index, body fat, trunk fat, SBP, DBP, and TyG-BMI. No significant association was identified between the tertiles of dairy consumption and other variables of body composition, anthropometric measurements, biochemical variables, and inflammatory biomarkers, outlined in Supplementary Table 1.

**Table 3:** CVD risk factors across tertiles of dairy consumption (*n* = 390)

Variables		Dairy Tertile			P-value
		T1	T2	T3	
		< 257.042 g	257.042–420.041 g	> 420.041 g	
Body mass index (Kg/m^2^)	**Crude**	31.43 ± 4.43	31.62 ± 4.52	30.77 ± 3.92	0.249
	**Model 1**	31.05 ± 0.53	31.86 ± 0.050	29.80 ± 0.49	**0.016**
	**Model 2**	31.19 ± 0.53	31.78 ± 0.49	29.77 ± 0.48	**0.016**
Fatty mas index	**Crude**	13.48 ± 3.38	13.86 ± 3.66	13.01 ± 3.11	0.135
	**Model 1**	13.18 ± 0.43	13.69 ± 0.40	12.09 ± 0.39	**0.019**
	**Model 2**	13.27 ± 0.43	13.63 ± 0.39	12.07 ± 0.39	**0.019**
Body fat (%)	**Crude**	42.38 ± 5.20	42.89 ± 5.62	41.43 ± 5.62	0.096
	**Model 1**	41.99 ± 0.71	42.22 ± 0.67	39.97 ± 0.65	**0.036**
	**Model 2**	42.07 ± 0.71	42.13 ± 0.66	39.98 ± 0.64	**0.039**
Body fat (Kg)	**Crude**	34.75 ± 8.719	35.63 ± 9.40	33.84 ± 8.05	0.258
	**Model 1**	33.65 ± 1.12	35.55 ± 1.05	31.59 ± 1.02	**0.032**
	**Model 2**	33.85 ± 1.12	35.38 ± 1.04	31.58 ± 1.01	**0.037**
Trunk fat (kg)	**Crude**	16.79 ± 3.57	17.16 ± 3.75	16.63 ± 3.71	0.495
	**Model 1**	16.25 ± 0.47	17.22 ± 0.44	15.57 ± 0.43	**0.034**
	**Model 2**	16.30 ± 0.47	17.15 ± 0.43	15.59 ± 0.42	**0.045**
Trunk fat (%)	**Crude**	321.36 ± 68.52	327.42 ± 73.05	312.81 ± 68.31	0.240
	**Model 1**	314.41 ± 8.90	326.41 ± 8.35	293.01 ± 8.10	**0.018**
	**Model 2**	315.85 ± 8.89	325.06 ± 8.21	293.10 ± 8.03	**0.021**
SBP (mmHg)	**Crude**	113.85 ± 14.79	113.97 ± 12.47	107.01 ± 16.13	**0.001**
	**Model 1**	111.78 ± 1.74	114.04 ± 1.63	108.24 ± 1.58	**0.045**
	**Model 2**	111.07 ± 1.78	114.70 ± 1.61	108.08 ± 1.56	**0.016**
DBP (mmHg)	**Crude**	78.64 ± 9.31	78.98 ± 10.07	75.29 ± 11.33	**0.023**
	**Model 1**	77.82 ± 1.24	79.44 ± 1.16	76.03 ± 1.13	0.127
	**Model 2**	77.03 ± 1.25	79.85 ± 1.13	76.18 ± 1.09	**0.062**
TyG-BMI	**Crude**	265.23 ± 48.54	261.96 ± 42.41	253.47 ± 36.05	0.185
	**Model 1**	258.17 ± 5.95	265.64 ± 5.48	248.41 ± 5.35	**0.070**
	**Model 2**	259.20 ± 6.01	265.17 ± 5.45	248.04 ± 5.34	**0.056**

Data as presented as mean ± standard deviation (SD). Crude p-values were obtained through One-way ANOVA. Other p-values were obtained through ANCOVA test. Bold valued indicates presence of statistical significance (P-value < 0.05) or marginally significance (p-value = 0.06 and 0.07)

**Model 1**: Adjusted for age, energy intake, BMI, physical activity, supplement

**Model 2**: Adjusted for age, energy intake, BMI, physical activity, supplement use, vegetables, meat, refined grain. BMI consider as collinear variable

### Associations between dairy tertiles and CVD risk factors

Table 4 presents crude and adjusted β-values, and 95% CI of the CVD risk factors, categorized by tertiles of dairy consumption. In comparison with the lowest tertile of dairy intake (< 257.042 g), consuming dairy between 257.042 and 420.041 g exhibited a positive association with fat-free mass (kg) and skeletal muscle mass (kg), irrespective of confounding variables. Conversely, higher dairy consumption (> 420.041 g, third tertile) demonstrated a negative association with adiposity markers such as BMI, fat mass index, and body fat compared to lower consumptions (< 257,042 g, first tertile), after controlling for confounders. However, a significant trend towards increased trunk fat (%) was observed with higher tertiles of dairy consumption.

**Table 4:** Associations between dairy tertiles and CVD risk factors (*n* = 390)

Variables		Dairy Tertile			P-trend
		T1	T2	T3	
		< 257.042 g	257.042–420.041 g	> 420.041 g	
Fat-free mass (kg)	**Crude**	**Reference**	0.11 (-1.26, 1.49)	0.25 (-1.27, 1.63)	0.719
	**Model 1**		**2.15 (0.12, 4.17)**	1.37 (-0.63, 3.36)	0.206
	**Model 2**		**2.02 (0.00, 4.05)**	1.32 (-0.70, 3.34)	0.253
Skeletal muscle mass (kg)	**Crude**		-0.08 (-0.91, 0.75)	0.05 (-0.78, 0.88)	0.901
	**Model 1**		**1.23 (0.04, 2.43)**	0.88 (-0.31, 2.04)	0.172
	**Model 2**		1.14 (-0.05, 2.33)	0.81 (-0.38, 2.00)	0.210
Body mass index (kg/m²)	**Crude**		0.19 (-0.85, 1.24)	-0.65 (-1.70, 0.39)	0.218
	**Model 1**		0.82 (-0.61, 2.25)	**-1.25 (-2.66, 0.16)**	0.810
	**Model 2**		0.59 (-0.83, 2.00)	**-1.42 (-2.83, -0.01)**	**0.038**
Fat mass index	**Crude**		0.37 (-0.45, 1.20)	-0.47 (-1.30, 0.36)	0.266
	**Model 1**		0.51 (-0.63, 1.65)	**-1.09 (-2.22, 0.04)**	0.051
	**Model 2**		0.35 (-0.78, 1.48)	**-1.20 (-2.33, -0.07)**	**0.031**
Body fat (%)	**Crude**		0.50 (-0.83, 1.84)	-0.95 (-2.28, 0.38)	0.161
	**Model 1**		0.23 (-1.66, 2.12)	**-2.02 (-3.89, -0.15)**	0.327
	**Model 2**		0.06 (-1.82, 1.94)	**-2.09 (-3.97, -0.21)**	**0.024**
Body fat (kg)	**Crude**		0.88 (-1.24, 3.00)	-0.91 (-3.03, 1.21)	0.399
	**Model 1**		1.90 (-1.08, 4.87)	-2.06 (-4.99, 0.88)	0.147
	**Model 2**		1.53 (-1.42, 4.48)	**-2.28 (-5.22, -0.67)**	0.067
Trunk fat (%)	**Crude**		6.07 (-10.92, 23.05)	-8.55 (-25.54, 8.43)	0.323
	**Model 1**		12.00 (-11.61, 35.61)	21.40 (-44.70, 1.89)	0.061
	**Model 2**		9.21(-14.10, 32.52)	22.75 (-45.99, 0.50)	**0.045**
WC (cm)	**Crude**		-3.02 (-7.51, 1.47)	-2.24 (-6.66, 2.18)	0.330
	**Model 1**		1.32 (-7.43, 10.07)	-2.81 (-11.00, 5.38)	0.450
	**Model 2**		0.46 (-8.27, 9.20)	-3.53 (-11.65, 4.59)	**0.030**
SBP (mmHg)	**Crude**		0.12 (-4.18, 4.42)	**-6.84 (-10.99, -2.68)**	**0.001**
	**Model 1**		2.26 (-2.35, 6.87)	**-3.55 (-8.08, -0.99)**	0.053
	**Model 2**		3.22 (-1.39, 7.83)	-2.20 (-6.81, 2.40)	0.298
DBP (mmHg)	**Crude**		0.34 (-2.70, 3.39)	**-3.35 (-0.41, 4.98)**	**0.020**
	**Model 1**		1.62 (-1.68, 4.91)	-1.79 (-5.03, 1.45)	0.256
	**Model 2**		2.64 (-0.63, 5.90)	-0.47 (-3.73, 2.79)	0.059
Fasting blood glucose (mg/dl)	**Crude**		-0.25 (-3.25, 2.74)	**-2.98 (-5.87, -0.10)**	**0.040**
	**Model 1**		1.52 (-2.08, 5,13)	-1.02 (-4.60, 2.56)	0.154
	**Model 2**		1.65 (-1.98, 5.28)	-0.94 (-4.59, 2.71)	0.575
TyG-BMI	**Crude**		-3.26 (-16.55, 10.03)	-11.76 (-24.65, 1.13)	0.072
	**Model 1**		7.47 (-8.09, 23.03)	-9.76 (-25.19, 5.67)	0.088
	**Model 2**		5.97 (-9.55, 21.49)	-11.15 (-26.67, 0.37)	**0.042**
TyG-WC	**Crude**		-43.92 (-102.30, 14.47)	-41.89 (96.73, 12.95)	0.150
	**Model 1**		11.07 (-71.38, 93.53)	-23.72 (-101.01, 53.57)	0.101
	**Model 2**		3.14 (-79.10, 85.37)	**-29.38 (-105.57, -16.81)**	**0.031**
PAI-1 (mg/dl)	**Crude**		3.14 (-8.41, 14.70)	9.55 (-1.24, 20.34)	0.078
	**Model 1**		-0.47 (-17.27, 16.32)	10.82 (-4.44, 26.09)	0.144
	**Model 2**		-3.76 (-20.41, 12.88)	8.59 (-6.57, 23.76)	**0.041**
TGF (ng/ml)	**Crude**		**19.76 (0.94, 38.58)**	6.78 (-10.89, 24.16)	0.508
	**Model 1**		20.71 (0.66, 40.76)	1.62 (-16.06, 19.30)	0.920
	**Model 2**		20.51 (0.29, 40.73)	2.00 (-15.83, 19.84)	0.901
IL-Iβ	**Crude**		-0.07 (-0.57, 0.43)	-0.07 (-0.59, 0.45)	0.788
	**Model 1**		-0.16 (-0.78, 0.45)	-0.28 (-0.89, 0.33)	0.369
	**Model 2**		-0.18 (-0.77, 0.40)	**-0.47 (-1.08, -0.14)**	0.500

Data are presented as β-value, 95% confidence interval (CI), and p-value for trend obtained through Linear regression. Bold valued indicates presence of statistical significance (P-value < 0.05) or marginally significance (p-value = 0.06 and 0.07)

Model 1: Adjusted for age, energy intake, BMI, physical activity, supplement

Model 2: Adjusted for age, energy intake, BMI, physical activity, supplement, vegetables, meat, refined grain

Abbreviation: BMI: body mass index; DBP: Diastolic blood pressure; IL-1β: Interleukin-1 β; PAI-1: Plasminogen activator inhibitor-1; SBP: Systolic blood pressure; TGF: Transforming growth factor; TyG index: Triglyceride-glucose index; WC, waist cirfumference

In the crude model, there was a significant negative association between SBP and higher dairy consumption (β: -6.84; 95% CI: -10.99, -2.68; *P* = 0.001), and a significant decreasing trend as tertiles increased (*P* = 0.001). After adjusting for confounders, the negative association between SBP and the third dairy tertile persisted (β: -3.55; 95% CI: -8.08, -0.99; *P* = 0.045). Regarding DBP, there was a consistent pattern of a significant decline in DBP corresponding to the rise in dairy tertiles in the unadjusted model.

Greater dairy product consumption (> 420,041 g) in the crude model exhibited a negative association with FBG compared to the lowest dairy consumption (β: -2.98; 95% CI: -5.87, -0.10; *P* = 0.043). The markers TyG-WC and IL-Iβ demonstrated a negative association with higher dairy consumption compared to lower consumption, regardless of confounders. Furthermore, a trend of decreasing TyG-BMI and TyG-WC was observed with increasing tertiles of dairy consumption, after controlling for confounders. No association was observed between the tertiles of dairy consumption and the variables presented in Supplementary Table 2.

### Association between dairy tertiles and ASCVD lifetime odds

**Fig. 1:**
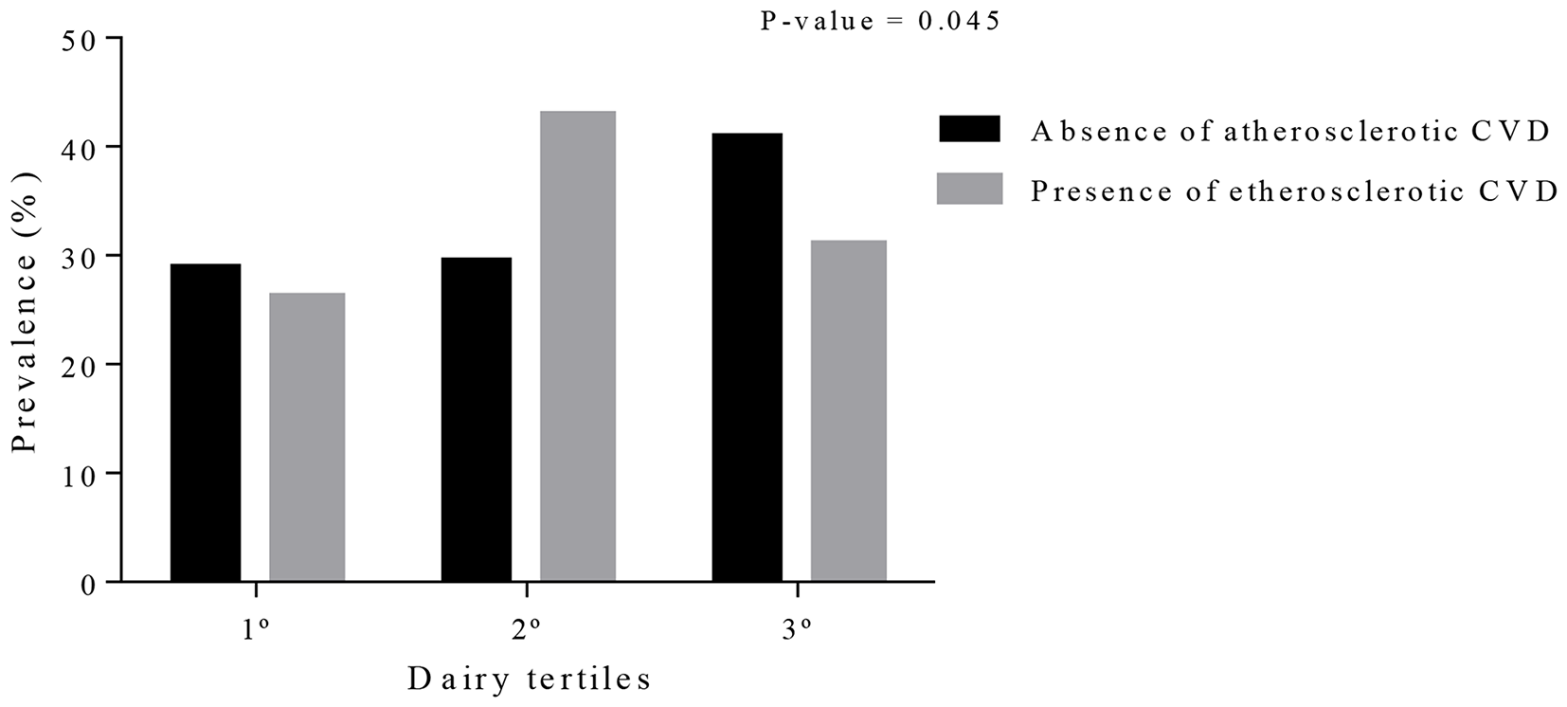
Prevalence of women with and without atherosclerotic cardiovascular disease (ASCVD) according to dairy tertiles. P-value was obtained through Chi-square test

The association between ASCVD lifetime odds and dairy tertiles among women with overweight and obesity is shown in Fig. 1 and Table 5. The prevalence of women without ASCVD increased according to dairy tertiles. In line with these findings, an association was observed between the consumption of dairy tertiles and ASCVD in the regression analysis. Only in the crude model, women in the third tertile of dairy consumption had a 0.79 lower chance of developing ASCVD compared with those in the first tertile of dairy consumption.

**Table 5:** Association between dairy tertiles and ASCVD lifetime odds (*n* = 390)

Outcome variable		Dairy tertiles	OR (95% CI)	*P*-value	*P*-trend
Atherosclerotic CVD lifetime	**Crude**	T2	0.84 (0.35, 2.01)	0.692	0.606
		T3	**0.21 (0.11, 0.41)**	**0.001**	
	**Model 1**	T2	3.39(0.86, 13.41)	0.082	0.601
		T3	1.65 (0.38, 7.17)	0.501	
	**Model 2**	T2	3.53 (0.82, 15.14)	0.090	0.593
		T3	1.80 (0.37, 8.60)	0.464	

Data are presented as odds ratio (OR), 95% confidence interval (CI), p-value, and p-value for trend were obtained through binary logistic regression. Bold valued indicates presence of statistical significance (P-value < 0.05)

Adjusted model 1: Adjusted for age, energy intake, BMI, physical activity, supplement

Adjusted model 2: Adjusted for age, energy intake, BMI, physical activity, supplement, vegetables, meat, refined grain

BMI was considered as collinear variable

## Discussion

In our crude model, we found that overweight and obese women who consumed more than (> 420,041 g) of dairy per week had a lower risk of ASCVD than those who consumed less than (< 257,042 g). However, this association was not significant after adjusting for potential confounders such as age, energy intake, BMI, physical activity, supplements, vegetables, meat, and whole grains. Moreover, we observed that high dairy consumption was inversely related to adiposity markers and blood pressure.

This cross-sectional study, which recruited 390 women with obesity and overweight, showed that higher dairy intake corresponded to lowers mean values of BMI, fat mass index, body fat, trunk fat, SBP, DBP, and TyG-BMI.

A meta-analysis of 22 prospective cohort studies reported an inverse association between dairy intake and the overall risk of CVD [57]. Contrary to our study, He et al. (2020) investigated the association between high milk consumption and carotid atherosclerosis in middle- and old-aged Chinese. Their findings indicated a significant association between higher milk consumption and an increased chance of carotid atherosclerosis [58]. These conflicting results might be due to the difference in our study populations. Our study focused on subjects aged 18 to 48 years with overweight and obesity, while study participants in the He et al. (2020) study were 50 years or older. Another disparity lies in the scope of assessment; He et al.’s study only assessed milk consumption, whereas our study considered various forms of dairy consumption. Furthermore, genetic variations between populations likely contribute to the observed distinctions.

In the current study, we noted a significant inverse association between increased dairy consumption and several variables, including BMI, WC, fat mass index, body fat, SBP, DBP, and TyG-BMI. This aligns with the findings of Azadbakht et al., who also observed an inverse association between dairy consumption and elevated blood pressure [59]. Aljuraiban et al. (2018) found that lower SBP and DBP were linked to increased consumption of low-fat dairy products [60]. There isn’t much information on the possible processes by which low-fat dairy products could raise blood pressure [61, 62]. The potential mechanism behind this association involves the angiotensin-converting enzyme-inhibitory effect of milk proteins, specifically within the renin-angiotensin system. This inhibition in adipocytes can mitigate both obesity and hypertension [63]. Other researchs indicates that dairy protein’s bioactive peptides are released by digesting enzymes and modify endothelium function, causing vasodilatation [64]. Additionally, measurements of arterial stiffness as determined by pulse wave velocity and dairy product intake appeared to be inversely correlated, according to the Maine Syracuse Longitudinal Study [65].

A RCT involving 34 adult participants observed a significant decrease in both body weight and body fat when individuals consumed three servings of fat-free yogurt per day [66]. Higher overall dairy consumption was linked to decreased weight gain in one study [67]. Another study showed that greater reductions in fat mass were observed with increased dairy intake. Adipocyte lipid metabolism, fat oxidation, fatty acid absorption, and postprandial fat metabolism have all been linked to calcium. Moreover, some research indicates dairy ingredients other than calcium, like proteins, medium-chain fatty acids, and conjugated linoleic acid, might be important [68]. Proteins, vitamin D, calcium, and phosphorus are among the elements found in dairy products that may help reduce weight gain and the chance of becoming overweight or obese [69, 70]. Various proposed mechanisms suggest that the positive impact of dairy consumption on weight could be attributed to increased calcium intake, leading to a reduction in lipogenesis and stimulation of lipolysis. This effect is likely due to the suppression of 1,25-dihydroxy vitamin D formation and the secretion of calciotropic and parathyroid hormones [71]. Apart from calcium, other components in dairy may contribute to the observed benefits in terms of body weight and fat loss [72]. Literature indicates that milk contains bioactive peptides, which may independently regulate body fat accumulation, acting beyond the influence of calcium [63, 73]. These bioactive peptides have been found to inhibit the angiotensin-converting enzyme, subsequently reducing the production of the angiotensin II hormone and leading to a decrease in fat deposition. Furthermore, whey protein, naturally present in milk, plays a role in controlling glucose metabolism in insulin-resistant individuals. It also enhances satiety by increasing the release of anorectic gut hormones like leptin and GLP-1, while decreasing the release of the orexigenic hormone ghrelin [74–76]. This contributes to a higher likelihood of both weight maintenance and weight loss. Additionally, conjugated linolenic acid, a family of fatty acids found in dairy foods, may regulate adipogenesis, inflammation, and lipid metabolism, exerting anti-obesity effects [77]. Regular dairy product consumption has been linked to a lower incidence of triglycerides [78]. The makeup of the gut microbiota could be a factor in the inverse relationship shown between yogurt consumption and hypertriglyceridemia. Commensal bacteria like Lactobacillus and Bifidobacterium are typically found in yogurt [79]. It has been demonstrated that intestinal microbiomes utilise dietary fibre and polyunsaturated fatty acids (PUFA) to create conjugated linoleic acid (CLA) and short-chain fatty acids (SCFAs). These acids have been linked to increased lipolysis and TG levels as well as higher clearance of very low-density lipids (VLDLs) and interactions with peroxisome proliferator-activated receptors (PPARs) [80]. Highlighting the benefits of both carbohydrates (lactose) and protein (whey and casein), milk is considered a healthy alternative to energy-dense beverages. Its consumption may reduce hunger and enhance adherence to a healthy diet [72]. In terms of WC, we saw that dairy consumption could impact on it. Based on studies, it was found that higher milk consumption can cause decrease in WC [81]. The mechanism by which dairy consumption impact on WC is as follow. Cortisone is converted into cortisol with the aid of 11-b-hydroxysteroid dehydrogenase-1. Calcitriol induces the expression of this enzyme. The content of calcitriol can rise due to insufficient calcium consumption. As a result, there is an increase in cortisol synthesis and fat storage, particularly around the abdomen [82].

In the current study, individuals in the higher tertiles of dairy consumption demonstrated elevated intakes of protein, cholesterol, fats, as well as vitamins A, D, E, B2, B5, B6, B12, sodium, potassium, magnesium, calcium, phosphorus, and zinc. A study by Young et al. revealed that heightened plasma potassium levels inhibit the formation of free radicals and the proliferation of vascular smooth muscle cells, along with reducing arterial thrombosis [83]. Establishing a direct association between a specific mineral in dairy products and hypertension proves challenging due to the crucial metabolic balance required among calcium, magnesium, and potassium. Strong correlations exist between the intakes of these minerals when dairy products are consumed. Notably, dairy products are significant sources of all three minerals. Additionally, milk, being a low-sodium food, provides an added advantage in reducing blood pressure [59]. Calcium intake can influence body fat mass through various mechanisms. Its simplest effect involves inhibiting the absorption of fat and fatty acids [84]. Calcium’s primary impact appears to be mediated by its influence on intracellular calcium control. Evidence suggests that the agouti gene products, expressed in human adipocytes, enhance calcium current into the cells. This, in turn, concurrently affects lipolysis and lipogenesis, leading to fat deposition in adipocytes. The product increases the activity of fatty acid synthetase and inhibits lipolysis through a calcium-dependent mechanism [85]. Calcitriol, which inhibits lipolysis, reduces the entry of calcium into cells. Higher calcium intake decreases the entry of calcium into cells by lowering concentrations of 1,25-dihydroxy vitamin D. Consequently, it inhibits fatty acid synthesis and promotes lipolytic activity. The beneficial effect of calcium in preventing fat accumulation can also be attributed to the expression of uncoupling protein 2 in white adipose tissue, contributing to thermogenesis [86]. A recent systematic review investigated the associations between biomarkers of dairy fat intake (pentadecanoic acid, heptadecanoic acid, trans-palmitoleic acid) and the risk of CVD. The review suggested that circulating levels of these diary fat biomarkers were not associated with an increased risk of CVD [87].

The present study has several strengths. Firstly, to the best of our knowledge, this is the first study investigating the associations between dairy consumption and the chance of ASCVD and its risk factors among Iranian women with overweight and obesity. Secondly, the assessment of dietary intake was conducted using a validated questionnaire. Finally, the study encompassed all dairy products consumed by the Iranian population.

However, there are several limitations to this study. It is an observational study, thereby limiting the ability to make causal inferences. Additionally, potential errors may be present in the dietary assessment due to recall bias and misclassification errors. Furthermore, the analysis considered total dairy consumption without examining individual foods or food groups separately.

## Conclusion

In conclusion, our findings initially indicated a lower likelihood of ASCVD in women with higher dairy consumption compared to those with lower consumption. However, this association diminished after adjusting for various potential confounding factors. In addition, a significant negative association was observed between dairy consumption and BMI, fat mass index, body fat, blood pressure, and TyG-BMI. The results of our study imply a protective association between dairy consumption and markers of adiposity, blood pressure, and ASCVD. We suggest further research to validate the findings of this study.

### Electronic supplementary material

Below is the link to the electronic supplementary material.

Supplementary Material 1

## Data Availability

The data that support the findings of this study are available from Khadijeh Mirzaei but restrictions apply to the availability of these data, which were used under license for the current study, and so are not publicly available. Data are however available from the authors upon reasonable request and with permission of Khadijeh Mirzaei.

## Abbreviations

CVD: Cardiovascular Disease
WHO: World Health Organization
FFQ: Food Frequency Questionnaire
BMI: Body Mass Index
SD: Standard Deviation
WC: Waist Circumference
WHtR: Waist-to-height Ratio
DASH: Dietary Approaches to Stop Hypertension
PCOS: Polycystic Ovary Syndrome
NAFLD: Non-alcoholic Fatty Liver Disease
TG: Triglyceride
TC: Total Cholesterol
LDL: Low-Density Lipoprotein
HDL: High-Density Lipoprotein
BIA: Bioimpedance Analysis
IPAC: International Physical Activity Questionnaire
SBP: Systolic Blood Pressure
DBP: Diastolic Blood Pressure
SFA: Saturated Fatty Acid
MUFA: Monounsaturated Fatty Acid
PUFA: Polyunsaturated Fatty Acid
ANOVA: Analysis of Variance
ANCOVA: Analysis of Covariance
GLM: Generalized Linear Models
CI: Confidence Interval
OR: Odds Ratio
