# Supplementary material for: Dairy intake and cardiovascular diseases risk factors: a cross-sectional study on Iranian obese and overweight women
Source: BMC Public Health. 2024 Jul 15;24:1895. doi: 10.1186/s12889-024-19232-z (PMC11251318; doi:10.1186/s12889-024-19232-z)
Supplement: Supplementary file 1 — Supplementary Material 1 [file 12889_2024_19232_MOESM1_ESM.docx]

**Supplementary Table 1:** Body composition, anthropometric measurements, and clinical markers according to dairy consumption tertiles (n=390).

| **Variables** | | Dairy Tertile | | | **P-value** |
| --- | --- | --- | --- | --- | --- |
|  |  | T1 | T2 | T3 |  |
|  |  | **<**257.042**g** | 257.042-420.041g | **>**420.041**g** |  |
| **Body Composition** | | | | | |
| Fat-free mass (Kg) | **Crude** | 46.37±5.84 | 46.48±6.00 | 46.62±5.18 | 0.938 |
|  | **Model 1** | 45.51±0.76 | 47.65±0.71 | 46.87±0.69 | 0.124 |
|  | **Model 2** | 45.57±0.77 | 47.59±0.71 | 46.88±0.69 | 0.163 |
| Skeletal muscle mass (Kg) | **Crude** | 25.55±3.61 | 25.47±3.57 | 25.60±3.07 | 0.952 |
|  | **Model 1** | 24.93±0.45 | 26.17±0.42 | 25.80±0.41 | 0.130 |
|  | **Model 2** | 24.98±0.45 | 26.12±0.42 | 25.79±0.41 | 0.182 |
| Fat-free mass index | **Crude** | 17.93±1.70 | 17.85±1.57 | 18.85±11.52 | 0.422 |
|  | **Model 1** | 17.82±0.18 | 18.17±0.17 | 17.82±0.17 | 0.271 |
|  | **Model 2** | 17.87±0.18 | 18.15±0.17 | 17.80±0.17 | 0.337 |
| Visceral fat area (cm^2^) | **Crude** | 165.52±39.78 | 183.80±149.56 | 164.42±38.14 | 0.165 |
|  | **Model 1** | 153.08±16.85 | 191.90±15.82 | 159.14±15.35 | 0.194 |
|  | **Model 2** | 153.50±17.21 | 191.08±15.90 | 159.57±15.54 | 0.222 |
| Visceral fat level (cm) | **Crude** | 17.32±17.32 | 16.23±3.25 | 16.73±12.15 | 0.776 |
|  | **Model 1** | 18.95±2.36 | 16.34±2.20 | 16.09±2.13 | 0.624 |
|  | **Model 2** | 19.00±2.41 | 16.20±2.21 | 16.18±2.15 | 0.627 |
| **Anthropometric measurements** | | | | | |
| Waist circumference (cm) | **Crude** | 99.20±10.89 | 96.17±16.66 | 96.96±17.52 | 0.396 |
|  |  |  |  |  |  |
|  | **Model 1** | 94.88±3.38 | 96.20±3.12 | 92.06±2.64 | 0.601 |
|  | **Model 2** | 95.46±3.42 | 95.92±3.13 | 91.92±2.63 | 0.577 |
| Waist-to-hip ratio | **Crude** | 0.93±0.05 | 1.63±7.98 | 0.93±0.05 | 0.371 |
|  | **Model 1** | 0.92±0.00 | 0.93±0.00 | 0.92±0.00 | 0.478 |
|  | **Model 2** | 0.92±0.00 | 0.93±0.00 | 0.92±0.00 | 0.433 |
| **Biochemical variables** | | | | | |
| HOMA_IR index | **Crude** | 3.46±1.37 | 3.21±1.22 | 3.31±1.23 | 0.501 |
|  | **Model 1** | 3.20±0.18 | 3.13±0.17 | 3.37±0.16 | 0.622 |
|  | **Model 2** | 3.14±0.19 | 3.15±0.17 | 3.40±0.16 | 0.507 |
| QUICKI (mg/l) | **Crude** | 0.49±0.02 | 0.49±0.02 | 0.49±0.02 | 0.889 |
|  | **Model 1** | 0.49±0.00 | 0.49±0.00 | 0.49±0.00 | 0.480 |
|  | **Model 2** | 0.49±0.00 | 0.50±0.00 | 0.49±0.00 | 0.555 |
| Fasting blood glucose (mg/dL) | **Crude** | 88.67±9.18 | 88.41±11.67 | 85.68±7.76 | 0.081 |
|  | **Model 1** | 86.96±1.38 | 88.48±1.28 | 85.94±1.24 | 0.386 |
|  | **Model 2** | 86.89±1.41 | 88.54±1.29 | 85.95±1.25 | 0.370 |
| Total cholesterol (mg/dL) | **Crude** | 189.73±37.31 | 184.20±37.02 | 181.28±34.23 | 0.313 |
|  | **Model 1** | 177.19±4.61 | 181.64±4.30 | 179.72±4.14 | 0.780 |
|  | **Model 2** | 176.96±4.74 | 181.73±4.34 | 179.81±4.22 | 0.762 |
| Triglycerides (mg/dL) | **Crude** | 122.69±62.11 | 114.21±57.07 | 116.95±60.04 | 0.665 |
|  | **Model 1** | 112.70±8.79 | 115.78±8.18 | 128.12±8.00 | 0.394 |
|  | **Model 2** | 111.62±8.94 | 115.75±8.17 | 129.00±8.06 | 0.329 |
| HDL (mg/dL) | **Crude** | 46.45±11.71 | 46.81±10.10 | 47.02±10.81 | 0.944 |
|  | **Model 1** | 47.28±1.43 | 46.62±1.33 | 46.35±1.28 | 0.890 |
|  | **Model 2** | 47.23±1.47 | 46.64±1.35 | 46.37±1.31 | 0.911 |
| LDL (mg/dL) | **Crude** | 95.24±23.93 | 96.84±23.77 | 92.86±24.70 | 0.565 |
|  | **Model 1** | 97.74±3.24 | 99.19±3.02 | 97.85±2.90 | 0.935 |
|  | **Model 2** | 97.78±3.32 | 99.16±3.04 | 97.85±2.96 | 0.940 |
| AST (IU/L) | **Crude** | 17.44±7.28 | 17.36±6.33 | 18.60±8.24 | 0.471 |
|  | **Model 1** | 17.64±1.10 | 17.83±1.03 | 18.13±0.99 | 0.947 |
|  | **Model 2** | 17.46±1.13 | 17.83±1.03 | 18.26±1.00 | 0.876 |
| ALT (IU/L) | **Crude** | 18.49±12.91 | 18.66±12.72 | 20.20±13.40 | 0.642 |
|  | **Model 1** | 19.21±2.02 | 19.55±1.88 | 19.95±1.81 | 0.964 |
|  | **Model 2** | 18.69±2.05 | 19.58±1.88 | 20.33±1.82 | 0.845 |
| **Atherogenic factors** | | | | | |
| Atherogenic index of plasma | **Crude** | 0.38±0.25 | 0.34±0.22 | 0.35±0.25 | 0.571 |
|  | **Model 1** | 0.34±0.03 | 0.35±0.03 | 0.39±0.03 | 0.613 |
|  | **Model 2** | 0.34±0.03 | 0.35±0.03 | 0.39±0.03 | 0.580 |
| Castelli’s risk index-2 | **Crude** | 4.40±2.07 | 4.08±1.20 | 4.01±1.08 | 0.224 |
|  | **Model 1** | 3.84±0.13 | 3.99±0.12 | 4.02±0.12 | 0.582 |
|  | **Model 2** | 3.83±0.13 | 3.99±0.12 | 4.02±0.12 | 0.581 |
| Castelli’s risk index-2 | **Crude** | 2.12±0.56 | 2.13±0.61 | 2.03±0.59 | 0.521 |
|  | **Model 1** | 2.12±0.08 | 2.18±0.08 | 2.18±0.07 | 0.836 |
|  | **Model 2** | 2.12±0.08 | 2.18±0.08 | 2.18±0.07 | 0.849 |
| Atherogenic coefficient | **Crude** | 3.40±2.07 | 3.08±1.20 | 3.01±1.08 | 0.224 |
|  | **Model 1** | 2.84±0.13 | 2.99±0.12 | 3.02±0.12 | 0.582 |
|  | **Model 2** | 2.83±0.13 | 2.99±0.12 | 3.02±0.12 | 0.581 |
| CHOLIndex | **Crude** | 48.78±21.45 | 50.02±22.45 | 45.84±23.46 | 0.466 |
|  | **Model 1** | 50.46±3.18 | 52.56±2.96 | 51.49±2.86 | 0.891 |
|  | **Model 2** | 50.54±3.26 | 52.51±2.99 | 51.47±2.90 | 0.906 |
| TyG index | **Crude** | 8.48±0.50 | 8.41±0.50 | 8.39±0.48 | 0.505 |
|  | **Model 1** | 8.40±0.07 | 8.42±0.06 | 8.47±0.06 | 0.736 |
|  | **Model 2** | 8.39±0.07 | 8.42±0.06 | 8.48±0.06 | 0.693 |
| TyG-Waist circumference | **Crude** | 835.67±133.13 | 791.75±144.72 | 793.78±169.88 | 0.244 |
|  | **Model 1** | 798.77±31.81 | 809.84±29.39 | 775.05±25.61 | 0.670 |
|  | **Model 2** | 803.68±32.00 | 806.82±29.44 | 774.30±25.43 | 0.660 |
| **Inflammatory biomarkers** | | | | | |
| PAI_1 (mg/dl) | **Crude** | 11.45±16.50 | 14.59±31.18 | 20.99±36.14 | 0.209 |
|  | **Model 1** | 12.82±6.36 | 12.35±6.01 | 23.64±5.07 | 0.282 |
|  | **Model 2** | 14.83±6.40 | 11.07±5.97 | 23.42±5.04 | 0.295 |
| Gal_3 (ng/ml) | **Crude** | 3.81±7.57 | 4.03±7.157 | 4.60±7.39 | 0.923 |
|  | **Model 1** | 5.50±2.27 | 3.75±2.45 | 6.01±2.05 | 0.801 |
|  | **Model 2** | 5.18±2.305 | 3.92±2.49 | 6.11±2.10 | 0.829 |
| MCP1 (mg/dl) | **Crude** | 40.34±72.99 | 52.86±94.37 | 58.30±105.51 | 0.478 |
|  | **Model 1** | 51.33±15.51 | 61.05±14.68 | 52.25±13.56 | 0.878 |
|  | **Model 2** | 54.69±16.03 | 60.57±14.85 | 50.21±13.84 | 0.885 |
| TGF (ng/ml) | **Crude** | 70.36±24.29 | 90.12±54.06 | 77.15±58.48 | 0.122 |
|  | **Model 1** | 70.80±7.11 | 91.52±7.66 | 72.42±6.13 | 0.112 |
|  | **Model 2** | 70.70±7.27 | 91.21±7.79 | 72.70±6.23 | 0.132 |
| IL_1β (ng/ml) | **Crude** | 2.75±0.86 | 2.68±0.95 | 2.68±1.04 | 0.955 |
|  | **Model 1** | 2.77±0.22 | 2.61±0.23 | 2.49±0.25 | 0.716 |
|  | **Model 2** | 2.83±0.22 | 2.65±0.22 | 2.36±0.26 | 0.417 |
| hs-CRP (mg/l) | **Crude** | 4.31±4.60 | 4.20±4.44 | 4.42±4.92 | 0.955 |
|  | **Model 1** | 4.58±0.69 | 4.17±0.65 | 4.82±0.62 | 0.783 |
|  | **Model 2** | 4.56±0.71 | 4.19±0.65 | 4.82±0.63 | 0.797 |

Data as presented as mean ± standard deviation (SD). Crude p-values were obtained through One-way ANOVA. Other p-values were obtained through ANCOVA test.

**Model 1:** Adjusted for age, energy intake, BMI, physical activity, supplement

**Model 2:** Adjusted for age, energy intake, BMI, physical activity, supplement, vegetables, meat, refined grain. BMI consider as collinear variable

ALT: Alanine aminotransferase; AST: Aspartate aminotransferase; Gal-3: Galactin-3; HDL: High density lipoprotein; HOMA-IR: Homeostatic model assessment for insulin resistance index; hs-CRP: high sensitive- C reactive protein; IL_1β: Interleukin-1 β; LDL: Low density lipoprotein; MCP-1: Monocyte chemoattractant protein-1; PAI-1: Plasminogen activator inhibitor-1; SBP: Systolic blood pressure; TGF: Transforming growth factor; TyG index: Triglyceride-glucose index.

**Supplementary Table 2.** Association between dairy tertiles with the lifetime atherosclerotic CVD in women with overweight and obesity (n=390).

| **Variables** | | **Dairy Tertile** | | | **P-trend** |
| --- | --- | --- | --- | --- | --- |
|  |  | **T1** | **T2** | **T3** |  |
|  |  | **<257.042g** | **257.042-420.041g** | **>420.041g** |  |
| Fat-free mass index | **Crude** |  | -0.08 (-1.73, 1.56) | 0.92 (-0.73, 2.56) | 0.277 |
|  | **Model 1** |  | 0.35 (-0.14, 0.85) | 0.00 (-0.49, 0.49) | 0.945 |
|  | **Model 2** |  | 0.28 (-0.21, 0.77) | -0.07 (-0.56, 0.42) | 0.731 |
| Trunk fat (kg) | **Crude** |  | 0.36 (-0.53, 1.26) | -0.17 (-1.06, 0.72) | 0.710 |
|  | **Model 1** |  | 0.96 (-0.29, 2.21) | -0.68 (-1.92, 0.55) | 0.243 |
|  | **Model 2** |  | 0.84 (-0.39, 2.08) | -0.71 (-1.94, 0.52) | 0.215 |
| Visceral fat area (cm^2^) | **Crude** |  | 18.28 (-4.07, 40.64) | -1.09 (-23.45, 21.26) | 0.921 |
|  | **Model 1** |  | 38.82 (-5.88, 83.53) | 6.07 (-38.04, 50.17) | 0.848 |
|  | **Model 2** |  | 37.58 (-7.52, 82.69) | 6.07 (-38.92, 51.06) | 0.866 |
| Visceral fat level | **Crude** |  | -1.09 (-4.09, 1.90) | -0.59 (-3.59, 2.40) | 0.701 |
|  | **Model 1** |  | -2.62 (-8.88, 3.64) | -2.87 (-9.03, 3.30) | 0.373 |
|  | **Model 2** |  | -2.80 (-9.10, 3.50) | -2.82 (-9.09, 0.45) | 0.394 |
| Waist-to-hip ratio | **Crude** |  | 0.702 (-0.42, 1.82) | 0.00 (-1.12, 1.12) | 0.997 |
|  | **Model 1** |  | 0.11 (-0.01, 0.03) | 0.00 (-0.01, 0.02) | 0.777 |
|  | **Model 2** |  | 0.01 (-0.01, 0.03) | 0.01 (-0.01, 0.02) | 0.536 |
| HOMA-IR | **Crude** |  | -0.25 (-0.65, 0.16) | -0.15 (-0.55, 0.25) | 0.482 |
|  | **Model 1** |  | -0.07 (-0.56, 0.42) | 0.17 (-0.31, 0.65) | 0.650 |
|  | **Model 2** |  | 0.01 (-0.49, 0.50) | 0.26 (-0.23, 0.75) | 0.286 |
| QUICKI (mg/l) | **Crude** |  | 0.00 (-0.01, 0.01) | 0.00 (-0.01, 0.01) | 0.685 |
|  | **Model 1** |  | 0.01 (0.00, 0.01) | 0.00 (-0.01, 0.01) | 0.880 |
|  | **Model 2** |  | 0.00 (0.00, 0.01) | 0.00 -0.01, 0.01) | 0.947 |
| Total cholesterol (mg/dl) | **Crude** |  | -5.53 (-16.80, 5.74) | -8.45 (-19.33, 2.43) | 0.130 |
|  | **Model 1** |  | 4.45 (-7.60, 16.52) | 2.53 (-9.45, 14.51) | 0.692 |
|  | **Model 2** |  | 4.77 (-7.44, 16.99) | 2.84 (-9.44, 15.13) | 0.670 |
| Triglycerides (mg/dl) | **Crude** |  | -8.49 (-27.21, 10.24) | -5.74 (-23.85, 12.37) | 0.547 |
|  | **Model 1** |  | 3.08 (-19.90, 26.07) | 15.42 (-7.49, 38.33) | 0.183 |
|  | **Model 2** |  | 4.13 (-18.89, 27.14) | 17.38 (-5.84, 40.60) | 0.137 |
| HDL (mg/dl) | **Crude** |  | 0.36 (-3.04, 3.76) | 0.57 (-2.72, 3.85) | 0.736 |
|  | **Model 1** |  | -0.65 (-4.39, 3.09) | -0.92 (-4.64, 2.80) | 0.630 |
|  | **Model 2** |  | -0.59 (-4.38, 3.20) | -0.86 (-4.67, 2.95) | 0.662 |
| LDL (mg/dl) | **Crude** |  | 1.60 (-5.94, 9.14) | -2.37 (-9.65, 4.90) | 0.505 |
|  | **Model 1** |  | 1.45 (-7.01, 9.91) | 0.12 (-8.28, 8.52) | 0.987 |
|  | **Model 2** |  | 1.38 (-7.18, 9.94) | 0.07 (-8.53, 8.68) | 0.999 |
| AST (IU/L) | **Crude** |  | -0.08 (-2.38, 2.22) | 1.16 (-1.06, 3.38) | 0.295 |
|  | **Model 1** |  | 0.19 (-2.71, 3.08) | 0.49 (-2.38, 3.36) | 0.737 |
|  | **Model 2** |  | 0.37 (-2.55, 3.28) | 0.80 (-2.13, 3.73) | 0.593 |
| ALT (IU/L) | **Crude** |  | 0.17 (-3.90, 4.24) | 1.71 (-2.22, 5.63) | 0.386 |
|  | **Model 1** |  | 0.34 (-4.94, 5.62) | 0.74 (-4.50, 5.98) | 0.781 |
|  | **Model 2** |  | 0.89 (-4.40, 6.17) | 1.64 (-3.68, 6.95) | 0.546 |
| Atherogenic index of plasma | **Crude** |  | -0.04 (-0.11, 0.04) | -0.03 (-0.11, 0.04) | 0.378 |
|  | **Model 1** |  | 0.00 (-0.09, 0.10) | 0.04 (-0.05, 0.14) | 0.345 |
|  | **Model 2** |  | 0.01 (-0.09, 0.10) | 0.05 (-0.05, 0.14) | 0.313 |
| Castelli index-1 | **Crude** |  | -0.32 (-0.79, 0.15) | -0.38 (-0.84, 0.07) | 0.101 |
|  | **Model 1** |  | 0.15 (-0.20, 0.50) | 0.18 (-0.17, 0.53) | 0.315 |
|  | **Model 2** |  | 0.15 (-0.20, 0.51) | 0.19 (-0.17, 0.55) | 0.309 |
| Castelli index-2 | **Crude** |  | 0.01 (-0.18, 0.19) | -0.08 (-0.26, 0.09) | 0.335 |
|  | **Model 1** |  | 0.06 (-0.16, 0.28) | 0.06 (-0.16, 0.28) | 0.582 |
|  | **Model 2** |  | 0.05 (-0.17, 0.28) | 0.06 (-0.16, 0.29) | 0.590 |
| Atherogenic coefficient | **Crude** |  | -0.32 (-0.79, 0.15) | -0.38 (-0.84, 0.07) | 0.101 |
|  | **Model 1** |  | 0.15 (-0.20, 0.50) | 0.18 (-0.17, 0.53) | 0.315 |
|  | **Model 2** |  | 0.15 (-0.20, 0.51) | 0.19 (-0.17, 0.55) | 0.309 |
| CHOLIndex (mmol/l) | **Crude** |  | 1.24 (-5.78, 8.26) | -2.94 (-9.72, 3.84) | 0.379 |
|  | **Model 1** |  | 2.10 (-6.21, 10.42) | 1.04 (-7.22, 9.29) | 0.816 |
|  | **Model 2** |  | 1.97 (-6.43, 10.37) | 0.93 (-7.52, 9.38) | 0.842 |
| TyG index | **Crude** |  | -0.07 (-0.22, 0.08) | -0.09 (-0.24, 0.06) | 0.264 |
|  | **Model 1** |  | 0.02 (-0.16, 0.20) | 0.07 (-0.11, 0.26) | 0.435 |
|  | **Model 2** |  | 0.02 (-0.16, 0.21) | 0.08 (-0.11, 0.27) | 0.385 |
| GAL-3 | **Crude** |  | 0.22 (-3.72, 4.15) | 0.79 (-3.11, 4.69) | 0.692 |
|  | **Model 1** |  | -1.75 (-7.92, 4.41) | 0.50 (-4.96, 5.97) | 0.862 |
|  | **Model 2** |  | -1.25 (-7.28, 4.77) | 0.93 (-4.51, 6.37) | 0.752 |
| MCP1 (mg/dl) | **Crude** |  | 12.53 (-17.99, 43.04) | 17.96 (-11.22, 47.14) | 0.232 |
|  | **Model 1** |  | 9.73 (-30.78, 50.24) | 0.92 (-39.15, 40.99) | 0.979 |
|  | **Model 2** |  | 5.89 (-35.33, 47.10) | -4.47 (-45.81, 36.87) | 0.815 |
| hsCRP (ml/l) | **Crude** |  | -0.11 (-1.60, 1.38) | 0.11 (-1.33, 1.55) | 0.871 |
|  | **Model 1** |  | -0.40 (-2.23, 1.42) | 0.24 (-1.56, 2.05) | 0.775 |
|  | **Model 2** |  | -0.37 (-2.21, 1.47) | 0.26 (-1.59, 2.11) | 0.761 |

Data are presented as β-value, 95% confidence interval (CI), and p-value for trend obtained through Linear regression. Bold valued indicates presence of statistical significance (P-value <0.05) or marginally significance (p-value = 0.06 and 0.07).

Model 1: Adjusted for age, energy intake, BMI, physical activity, supplement.

Model 2: Adjusted for age, energy intake, BMI, physical activity, supplement, vegetables, meat, refined grain.

ALT: Alanine aminotransferase; AST: Aspartate aminotransferase; Gal-3: Galactin-3; HC: HDL: High-density lipoprotein; HOMA-IR Index: Homeostatic model assessment for insulin resistance index; hs-CRP: high sensitive- C reactive protein; LDL: Low-density lipoprotein; MCP-1: Monocyte chemoattractant protein-1; QUICKI: quantitative insulin-sensitivity check index; TyG index: Triglyceride-glucose index.
